# A Case of Adherent Placenta

**Published:** 1890-03

**Authors:** Isham Harrison

**Affiliations:** Deerbrook, Miss.


### A CASE OF ADHERENT PLACENTA.

By ISHAM HARRISON, M. D., DEERBROOK, MISS.

A genuine case of morbidly adherent placenta in which the adhesion is so firm and complete that it is utterly impossible to detach it, and a large portion, from necessity, must be left in the uterus, is, indeed, a rare occurrence.

"The frequency of these," says Playfair, "have been overestimated. Many cases believed to be examples of adherent placentæ are, in reality, only cases of placentæ retained from uterine inertia." That cases corresponding with the above description do occur, however, no one will deny. Barnes, in his "Obstetric Operations," quotes the following passage from Ramsbotham, the truth of which he attests from his own experience: "Instances are sometimes met with in which a portion of placenta is so closely cemented to the uterine surface that it cannot, by any means, be detached; nay, I have opened more than one body where a part was left adherent to the uterus, and where, on making a longitudinal section of the organ and examining the cut edges, I could not determine the boundary line between the uterus and placenta, so intimate a union had taken place between them."

When a part of the placenta is left in the uterus the patient is exposed to the fearful hazards of exhausting secondary hemorrhages and septic infection. Such cases necessarily occasion the practitioner considerable trouble and anxie[illegible]. The cause of adhesion is claimed to be a "morbid state of the decidua," resulting from a diseased condition of the lining membrane of the uterus, existing before pregnancy. Many signs are offered by which an adherent placenta may be suspected, but the only means of a positive diagnosis is in the introduction of the hand into the uterus. Without further preliminary remarks I will proceed to give an account of a case which occurred in my practice.

Jan. 18th I was called to see Harriet B., colored, multipara, about 40 years of age, and rather fleshy. I arrived about 2 o'clock A. M. At 10 P. M., under the auspices of a granny, she had given birth to a full term child, in a partial state of decomposition, but the placenta refused to come away. I found, upon digital examination, that it was still *in utero*. After employing Credé's method, unsuccessfully, to promote its expulsion, I determined to introduce my hand into the uterus and remove it. The entire uterus was firmly contracted, but there was a tetanoid constriction at the *os internum* which, especially, offered the most stubborn resistance to dilation. I refer to this only incidentally, as I do not consider it, properly speaking, an "hour-glass contraction." Finally I succeeded in passing the tips of my fingers beyond the constriction to the lower placental border, and ascertained that the placenta was adherent. The uterine contraction was so difficult to overcome, and my efforts caused the patient such intolerable pain, that I decided to postpone further procedures until she could be thoroughly anæsthetized. Accordingly, I sent for Dr. W. W. Hamilton, who lived at some distance, to assist me. He arrived after 11 o'clock A. M., more than thirteen hours after the birth of the child. During this interval the patient was remarkably free from hemorrhage. Chloroform was administered, and, after persistent and prolonged effort, we succeeded in dilating the uterus with the fingers. The placenta was found to be so intimately adherent to the uterine wall throughout its entire extent, that the two structures seemed to be blended into one. The fingers could not be insinuated between the uterus and placenta at any point of the margin. Fearing that the use of too much force might do serious injury to the uterine structure, after tearing away about half of the placental mass, we thought it prudent to desist. The cavity of the uterus was then thoroughly disinfected with a solution of the permanganate of potassium, and vaginal injections of the same ordered to be given twice daily, with quinine and laxatives internally. Owing to the distance at which she lived and a pressure of business at the time, it was impossible to regularly administer intra-uterine injections. She did finely until the third day, when severe abdominal pains and tenderness developed, with a considerable rise of temperature. These symptoms, under appropriate treatment, soon disappeared, and afterwards the progress of her convalescence was uninterrupted. The placenta gradually disintegrated, small lumps and particles appearing after each vaginal injection. One feature of the case to which I would call attention is the fact that there was no hemorrhage at any time after our attempt to remove the placenta.

